# Feature-based ensemble modeling for addressing diabetes data imbalance using the SMOTE, RUS, and random forest methods: a prediction study

**DOI:** 10.12771/emj.2025.00353

**Published:** 2025-04-15

**Authors:** Younseo Jang

**Affiliations:** College of Medicine, Ewha Womans University, Seoul, Korea

**Keywords:** Area under curve, Computer neural networks, Deep learning, Diabetes mellitus, Random forest

## Abstract

**Purpose:**

This study developed and evaluated a feature-based ensemble model integrating the synthetic minority oversampling technique (SMOTE) and random undersampling (RUS) methods with a random forest approach to address class imbalance in machine learning for early diabetes detection, aiming to improve predictive performance.

**Methods:**

Using the Scikit-learn diabetes dataset (442 samples, 10 features), we binarized the target variable (diabetes progression) at the 75th percentile and split it 80:20 using stratified sampling. The training set was balanced to a 1:2 minority-to-majority ratio via SMOTE (0.6) and RUS (0.66). A feature-based ensemble model was constructed by training random forest classifiers on 10 two-feature subsets, selected based on feature importance, and combining their outputs using soft voting. Performance was compared against 13 baseline models, using accuracy and area under the curve (AUC) as metrics on the imbalanced test set.

**Results:**

The feature-based ensemble model and balanced random forest both achieved the highest accuracy (0.8764), followed by the fully connected neural network (0.8700). The ensemble model had an excellent AUC (0.9227), while k-nearest neighbors had the lowest accuracy (0.8427). Visualizations confirmed its superior discriminative ability, especially for the minority (high-risk) class, which is a critical factor in medical contexts.

**Conclusion:**

Integrating SMOTE, RUS, and feature-based ensemble learning improved classification performance in imbalanced diabetes datasets by delivering robust accuracy and high recall for the minority class. This approach outperforms traditional resampling techniques and deep learning models, offering a scalable and interpretable solution for early diabetes prediction and potentially other medical applications.

## Introduction

### Background

Diabetes mellitus is a chronic metabolic disorder characterized by persistent hyperglycemia resulting from impaired insulin secretion or action. It is a major global health concern, affecting millions worldwide and leading to serious complications such as cardiovascular disease, kidney failure, neuropathy, and retinopathy.

Early detection and prediction of diabetes are critical for effective disease management and prevention of complications. Machine learning models have increasingly been used to identify individuals at high risk of developing diabetes; however, these models often suffer from class imbalance. In many datasets, there are significantly fewer diagnosed diabetes cases than non-diabetic cases, resulting in biased predictions and reduced sensitivity for high-risk patients. Addressing this imbalance is essential to improve model accuracy and ensure reliable early detection.

Data imbalance is a significant challenge in machine learning because an unequal distribution of classes can lead to biased predictions. This issue is particularly critical in medical and financial applications, where misclassifying minority instances may have severe consequences [1]. Conventional machine learning models tend to favor the majority class, resulting in suboptimal recall for the minority class. Effectively mitigating this bias necessitates a strategy that enhances predictive performance without introducing new biases.

In traditional epidemiological studies, an imbalance between exposed and unexposed groups may be less problematic compared to the challenges posed by feature-based classification models in machine learning. This study emphasizes the importance of addressing class imbalance in machine learning-based classification models relative to traditional epidemiological approaches.

Existing solutions to data imbalance include oversampling techniques such as the synthetic minority oversampling technique (SMOTE) [2], undersampling methods like Tomek Links [3], and cost-sensitive learning [4]. Although SMOTE increases the minority class size without exact duplication—thereby helping models generalize better—it can introduce noise if synthetic samples overlap with the majority class regions, potentially confusing the classifier. The Tomek Links method may discard useful majority class data, especially in small datasets, and might not fully correct severe imbalance. Cost-sensitive learning requires domain-specific expertise to set appropriate costs, and poor cost choices can impair performance. Random undersampling (RUS) is a straightforward method that randomly removes samples from the majority class to balance the dataset with the minority class [5]; however, it risks eliminating valuable information and may lead to under-fitting when too much data is discarded. Overcoming these drawbacks is necessary to effectively resolve data imbalance.

### Objectives

This study proposes a method that integrates SMOTE and RUS with a feature-based ensemble learning approach using a random forest to improve classification performance while minimizing the inherent drawbacks of individual resampling techniques.

## Methods

### Ethics statement

This study is a secondary analysis of a publicly available database from scikit-learn (https://scikit-learn.org/). Institutional review board approval and informed consent were not required.

### Study design

This prediction study compares various models designed to address the data imbalance problem in machine learning research. The study was conducted according to the TRIPOD-AI reporting guidelines for articles on deep learning in the medical field (development or prediction), available at https://www.tripod-statement.org/.

### Setting/participants

Model training was conducted between January 2025 and March 2025 using the Scikit-learn diabetes dataset. Participant information in the dataset was de-identified.

### Data source

This study utilizes the diabetes dataset from Scikit-learn, which comprises 442 samples and 10 continuous features. The dataset has been standardized to a mean of 0 and a standard deviation of 1. The target variable represents diabetes progression 1 year post-diagnosis (Dataset 1).

**Fig. 1.**
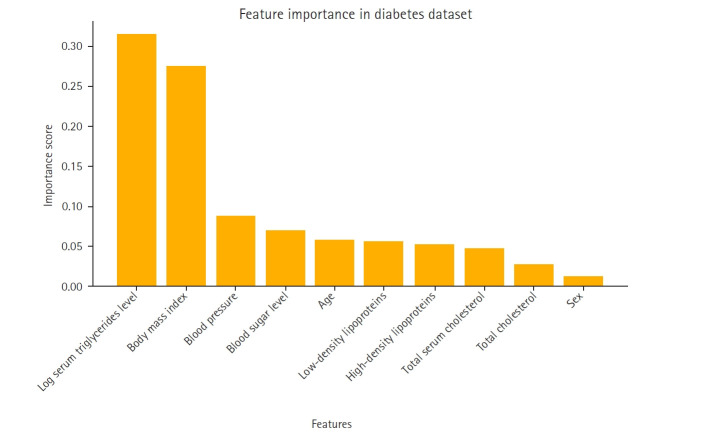
Feature importance in the Scikit-learn diabetes dataset.

A feature importance analysis was performed using the random forest algorithm to identify the most influential features for predicting diabetes progression [6]. The most significant predictors were log serum triglycerides level, body mass index, and blood pressure, while sex and high-density lipoproteins contributed the least to the model’s performance. The importance ranking of these features is visualized in Fig. 1.

The feature importance rankings in Fig. 1, computed using the mean decrease in impurity method in the random forest model, were instrumental in constructing the feature-based ensemble model. Specifically, 2-feature subsets were formed by prioritizing features with high importance scores. This strategy ensured that the ensemble model was built on the most informative feature combinations, thereby reducing noise and enhancing predictive performance. Fig. 1 not only displays the key variables that influence the model’s decision-making process but also supports the study’s methodological framework by guiding the feature selection process.

### Data preprocessing

The continuous target variable representing diabetes progression was transformed into a binary classification using the 75th percentile (Q3) as the threshold. Q3, the value below which 75% of the data fall, was used to label individuals with values above this threshold as high-risk (1) and those at or below it as low-risk (0). This binarization allowed the model to focus on identifying patients with the most severe progression of diabetes.

After binarization, the dataset was split into training and test sets in an 80:20 ratio using stratified sampling to preserve class distribution across both subsets. To prevent data leakage, SMOTE and RUS were applied exclusively to the training set after the split. The test set remained in its original, imbalanced state and was reserved solely for final evaluation. This strategy ensured that the model was trained on a balanced distribution while being evaluated on realistic, untouched data, thus preserving the validity of the performance metrics. All feature values were standardized using Z-score normalization, which improved the stability and convergence of the machine learning models.

To address class imbalance in the training set, SMOTE with a sampling strategy of 0.6 was first applied to generate synthetic samples for the minority class. Subsequently, RUS with a strategy of 0.66 reduced the number of majority class samples. The 0.66 ratio was chosen based on the new class distribution after applying SMOTE, ensuring that the final majority-to-minority ratio reached approximately 2:1. These resampling techniques resulted in a final class ratio of approximately 1:2 (minority:majority), achieving improved balance without excessively inflating the minority class or discarding too much majority class information.

Finally, the dataset was examined for missing values and extreme outliers. Since none were detected, no additional imputation or filtering was applied.

### Class distribution analysis

**Fig. 2.**
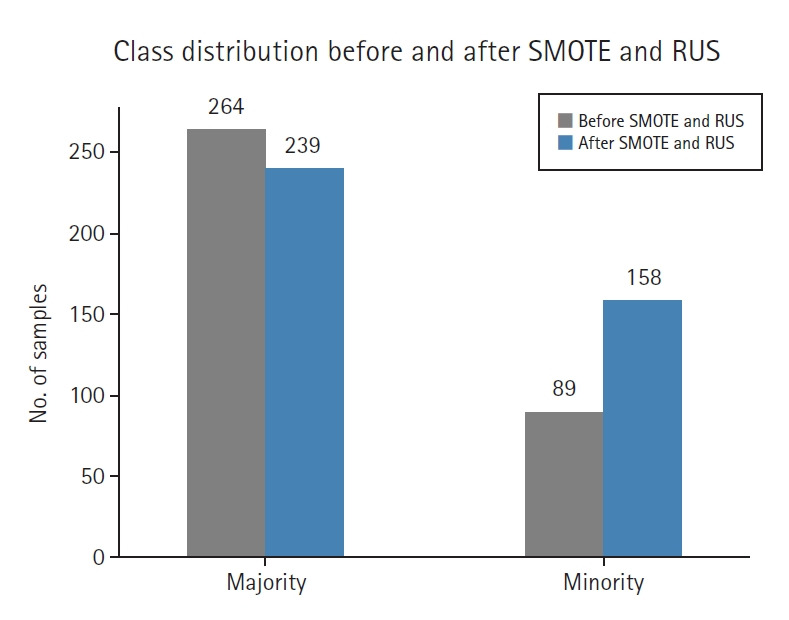
Class distribution before and after synthetic minority oversampling technique (SMOTE) and random undersampling (RUS).

Fig. 2 illustrates the impact of SMOTE and RUS on class balance. After resampling, the original imbalance was reduced from approximately 1:3.4 to a more balanced 1:2 ratio, leading to improved training data distribution.

Before resampling, the training set included 89 high-risk (minority) and 264 low-risk (majority) samples. After applying SMOTE (0.6) and RUS (0.66), the class distribution was adjusted to 158 high-risk and 239 low-risk samples, resulting in a 1:1.5 ratio.

### Outcome variables

Model outcomes were evaluated using the accuracy and area under the curve (AUC) metrics.

### Study size

All data in the diabetes dataset were utilized for training; no sample size estimation was performed.

### Feature-based ensemble modeling

To enhance interpretability and reduce overfitting, feature importance was first computed using a random forest classifier trained on the resampled dataset. It is important to note that the random forest used within the proposed feature-based ensemble model is conceptually distinct from the standalone random forest model used as a baseline. While the baseline random forest was trained on the original dataset using all features simultaneously, the ensemble model comprises multiple random forest classifiers trained on selected 2-feature subsets from the resampled (SMOTE+RUS) dataset.

Features were ranked based on their importance scores using the mean decrease in impurity criterion. The top-ranked features were then used to construct multiple 2-feature subsets. Based on these scores, the top 5 features were selected to form all possible pairwise combinations (n=10). Each 2-feature subset was used to train a separate random forest classifier, resulting in a total of 10 base learners.

These individual models were combined using a soft voting ensemble strategy [7]. Each model contributed its predicted class probabilities, which were averaged to yield the final classification. This soft voting approach, which averaged probabilities across all base learners and selecting the class with the highest average, enabled the ensemble to capture diverse patterns across feature pairs while maintaining robust generalization performance.

Random forest was chosen as the base classifier for several reasons. First, compared to deep learning models, random forest offers greater interpretability by providing feature importance analysis, which is particularly valuable in medical applications. Second, the small sample size (442 samples) and inherent class imbalance make the dataset prone to overfitting; random forest is known for its robustness under such conditions. Third, as an ensemble method itself, random forest works synergistically with the higher-level soft voting structure, contributing to stable and consistent performance.

SMOTE and RUS were applied solely to the training data, while the test data remained in its original, non-augmented form (with only standardization applied). This ensured that performance evaluations reflected the model’s ability to generalize to unseen, real-world distributions rather than to synthetic, balanced data—a crucial evaluation strategy to avoid overestimating model performance.

### Comparative models

Three resampling-based techniques were included for performance comparison: SmoteENN, balanced random forest, and SMOTE combined with Tomek Links. These methods are widely adopted to address class imbalance in medical data classification tasks due to their strong empirical performance and interpretability.

SmoteENN integrates oversampling with data cleaning by removing borderline and noisy samples after synthetic instances are generated [8]. A balanced random forest applies RUS to each bootstrap sample to maintain class balance within decision trees [7]. SMOTE+Tomek Links combines oversampling with a noise-reduction step that eliminates overlapping examples from different classes [9].

These 3 methods were implemented under the same experimental conditions as the other models, with the same Q3 binarization threshold, an 80:20 stratified split, and Z-score normalization applied consistently to ensure fairness in comparison. As a result, any observed performance differences can be attributed to the modeling strategies rather than preprocessing variations.

Furthermore, to evaluate the performance of the proposed feature-based ensemble model, we implemented 10 baseline classification models: logistic regression, random forest, gradient boosting, support vector machine (SVM), k-nearest neighbors (KNN), fully connected neural network (FCNN), deep neural network (DNN), recurrent neural network (RNN), long short-term memory, and wide & deep models.

Ten traditional deep learning and machine learning models were trained on the original, imbalanced dataset—without resampling (i.e., no SMOTE or RUS)—to assess their performance under realistic data conditions. Preprocessing included standard Z-score normalization, and the target variable was binarized using the Q3 (75th percentile) threshold consistently across all models.

For the deep learning models, architectures were designed using TensorFlow/Keras with common configurations (e.g., 2–3 dense layers with ReLU activations, dropout for regularization, and a sigmoid output layer). Training was conducted over 50–100 epochs with early stopping as appropriate. Traditional machine learning models were implemented using Scikit-learn with default hyperparameters, except for SVM, which utilized a radial basis function kernel and probability estimation.

Rather than training a single model on all features simultaneously, 2-feature subsets were created and separate random forest classifiers were trained on each subset. This technique reduced noise interference and mitigated overfitting while leveraging soft voting to aggregate predictions from individual models, thereby enhancing generalization.

### Evaluation metrics

Model performance was evaluated using accuracy and AUC, with a particular focus on the minority (high-risk) class—the primary interest of this study.

### Statistical methods

No additional statistical analyses were performed beyond the evaluation of accuracy and AUC.

## Results

### Model performance comparison

**Fig. 3.**
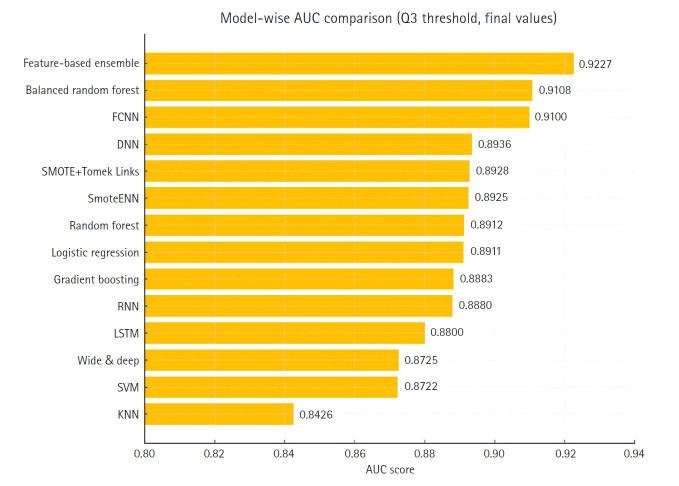
Comparison of the area under the curve across 14 models. AUC, area under the curve; FCNN, fully connected neural network; DNN, deep neural network; RNN, recurrent neural network; LSTM, long short-term memory; SVM, support vector machine; KNN, k-nearest neighbors.

Table 1 summarizes the accuracy scores of 14 different models. The feature-based ensemble model achieved an accuracy of 0.8764. Although accuracy alone may not fully capture model performance in imbalanced data, it remains a useful baseline metric for overall classification. Fig. 3 presents a horizontal bar chart comparing AUC scores across all models.

**Fig. 4.**
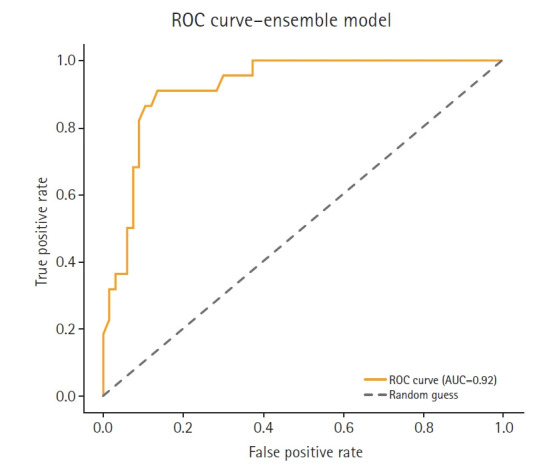
Receiver operating characteristic (ROC) curve of the feature-based ensemble modeling in this study. AUC, area under the curve.

**Fig. 5.**
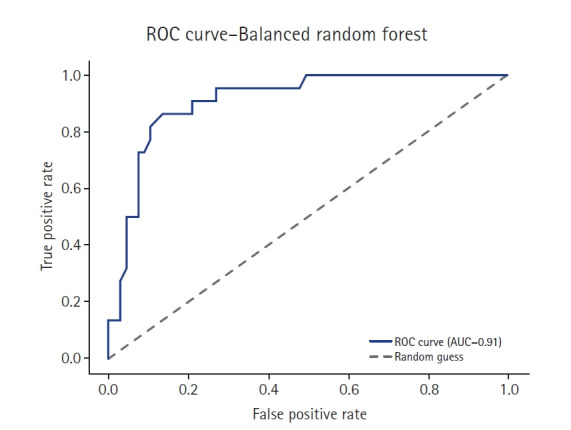
Receiver operating characteristic (ROC) curve of the balanced random forest model. AUC, area under the curve.

**Fig. 6.**
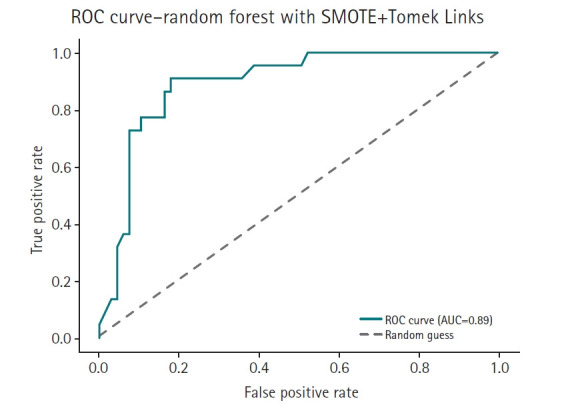
Receiver operating characteristic (ROC) curve of the SMOTE+Tomek Links model. AUC, area under the curve.

**Fig. 7.**
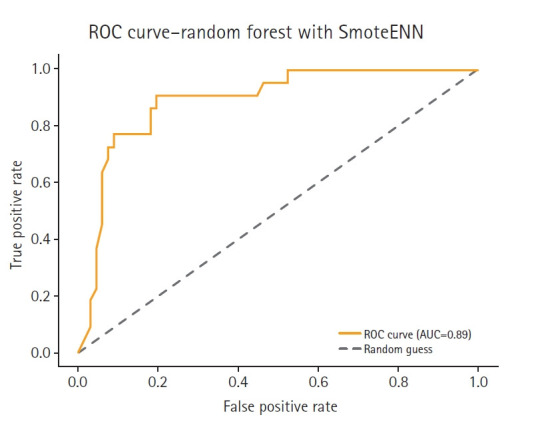
Receiver operating characteristic (ROC) curve of the SmoteENN model. AUC, area under the curve.

To complement the performance metrics summarized in Fig. 2, Figs. 4–7 and supplementary figures (Supplement 2) illustrate a model-wise comparison of AUC scores. This visualization provides an intuitive overview of each model’s discriminative ability, highlighting that the feature-based ensemble model achieved an AUC of 0.9227. Other models, such as balanced random forest, FCNN, and DNN, also demonstrated relatively high AUC values, while k-nearest neighbors showed the lowest. This figure reinforces the tabulated metrics by emphasizing the relative ranking in terms of AUC performance.

## Discussion

### Key results

The feature-based ensemble model and balanced random forest achieved the highest accuracy of 0.8764, followed by the fully connected neural network (0.8700) and SMOTE+Tomek Links (0.8652). Notably, the feature-based ensemble model demonstrated a superior AUC of 0.9227, confirming its effectiveness.

### Interpretation

The high AUC score further supports the ensemble model’s strong discriminative power, particularly in distinguishing high-risk individuals. These results underscore the effectiveness of integrating SMOTE, RUS, and feature-based ensemble modeling in enhancing both robustness and interpretability—especially in small, imbalanced medical datasets.

Although deep learning models such as DNN and RNN achieved relatively high AUC scores, they were not adopted as the primary approach in this study, owing to their requirement for large datasets and limited interpretability. In contrast, the random forest offers strong performance, robustness with small sample sizes, and useful feature importance analysis, making it more suitable for our proposed ensemble framework.

This study emphasized AUC due to its relevance to imbalanced classification problems. While accuracy can be misleading in skewed datasets, AUC captures the model’s ability to discriminate between classes across a range of thresholds, making it especially suitable for evaluating models in clinical prediction tasks where correctly identifying the minority (high-risk) class is crucial.

To validate the proposed method, we compared its performance against 3 widely adopted resampling-based approaches for imbalanced classification: SmoteENN, balanced random forest, and SMOTE combined with Tomek Links. These methods are frequently cited in recent literature and are considered standard benchmarks in medical data analysis.

Under identical preprocessing and evaluation conditions, the proposed feature-based ensemble model demonstrated comparable or superior performance relative to all 3 baseline methods. This suggests that the ensemble approach offers a compelling, empirically grounded alternative for handling class imbalance, particularly in the small and structured datasets common in healthcare applications.

### Comparison with previous studies

Previous studies addressing small data imbalance with the present model remain limited. Salmi et al. [1] noted in their review that, while deep learning is underexplored for structured medical data due to small sample sizes and model complexity, hybrid approaches combining sampling with machine learning models show promise. They discussed how techniques like SMOTE can augment small datasets, thereby improving performance in disease prediction tasks. Rather et al. [10] in 2024 examined how small, imbalanced datasets can benefit from fine-tuning pre-trained models, whereas this study leveraged the interpretability of the random forest. These findings align with the findings of Mujahid et al. [11] in 2024 on the efficacy of oversampling in small datasets. Their work compared oversampling techniques such as SMOTE, SVM-smote, borderline SMOTE, k-means SMOTE, and ADASYN on small, imbalanced datasets—for example, twitter sentiment data. Although not exclusively focused on deep learning, the study evaluated these methods with both machine learning and deep learning models and demonstrated improved performance on small datasets (e.g., hundreds of samples) after balancing. Overall, the findings suggest that oversampling enhances deep model consistency, particularly when sample sizes are limited, as validated through k-fold cross-validation.

### Strength

This study highlights the effectiveness of combining SMOTE, RUS, and feature-based ensemble learning in managing class imbalance. Unlike traditional methods, this approach improves minority class recall while maintaining overall model accuracy. These improvements are particularly valuable in domains where accurate predictions for the minority class are critical, such as in healthcare and fraud detection.

### Limitations

Despite its advantages, our approach has several limitations. First, the computational complexity increases due to the multiple model training steps, although overall performance remained comparable across models. Second, while the feature-based ensemble model enhances generalization, additional optimization is necessary to ensure efficiency when scaling to larger datasets. Furthermore, SMOTE may introduce synthetic samples that do not fully represent real data variations, potentially leading to model biases.

A critical concern is the risk of overfitting when an excessive number of synthetic samples are generated; if the minority class is expanded too much, the model may learn patterns that do not generalize well to unseen data, thus reducing real-world performance. To mitigate this issue, careful tuning of the sampling ratio and validation with independent datasets is essential.

### Clinical implications

The method proposed in this study, which integrates SMOTE, RUS, and feature-based ensemble learning, has proven effective in addressing class imbalance in diabetes prediction models. Moreover, this approach is applicable to other medical research fields beyond diabetes. For instance, in cancer prediction, early-stage cancer patients comprise only a small fraction of the total patient population, causing traditional machine learning models to struggle with accurate predictions. By applying the proposed method, the accuracy of predictions for early-stage cancer patients can be improved, thereby aiding early diagnosis.

### Suggestion for further studies

Future research should explore expanding the study to larger datasets and diverse application domains to validate the generalizability of the findings. Additionally, integrating hybrid approaches that combine cost-sensitive learning with ensemble modeling could further balance interpretability and performance.

### Conclusion

This study presents a novel approach that combines SMOTE, RUS, and feature-based ensemble learning using the random forest to address the data imbalance problem. Experimental results confirm that this approach significantly enhances predictive accuracy and recall for minority class instances while maintaining overall model robustness.

## Figures and Tables

**Table 1.** Comparison of accuracies of 14 models

Model	Accuracy
Feature-based ensemble model	0.8764
Balanced random forest	0.8764
Fully connected neural network	0.8700
SMOTE+Tomek Links	0.8652
K-nearest neighbors	0.8427
Logistic regression	0.8346
Support vector machine	0.8346
Long short-term memory	0.8300
Deep neural network	0.8202
Recurrent neural network	0.8202
SmoteENN	0.8202
Random forest	0.8195
Gradient boosting	0.8195
Wide & deep	0.8090

## Supplementary materials

Supplementary files are available from Harvard Dataverse: https://doi.org/10.7910/DVN/MVSFHY

Supplement 1. Python code for all the machine learning and deep learning methods. It is also available on GitHub, https://github.com/YounseoJang/diabetes-imbalance-ensemble.

Supplement 2. Receiver operating characteristic curves for the 10 traditional deep learning or machine learning models.
