# Supplementary material for: Feature-based ensemble modeling for addressing diabetes data imbalance using the SMOTE, RUS, and random forest methods: a prediction study
Source: Ewha Med J. 2025 Apr 15;48(2):e32. doi: 10.12771/emj.2025.00353 (PMC12277495; doi:10.12771/emj.2025.00353)

Supplement 2. Model-wise ROC Curve Comparisons

It provides the ROC curves for each classification model used in this study. These curves illustrate the trade-off between sensitivity (True Positive Rate) and specificity (False Positive Rate) for each model across different threshold levels. AUC scores are indicated in the legends of each plot to highlight the discriminative performance of each classifier. Only traditional machine learning and deep learning models were included.

ROC Curve - Logistic Regression

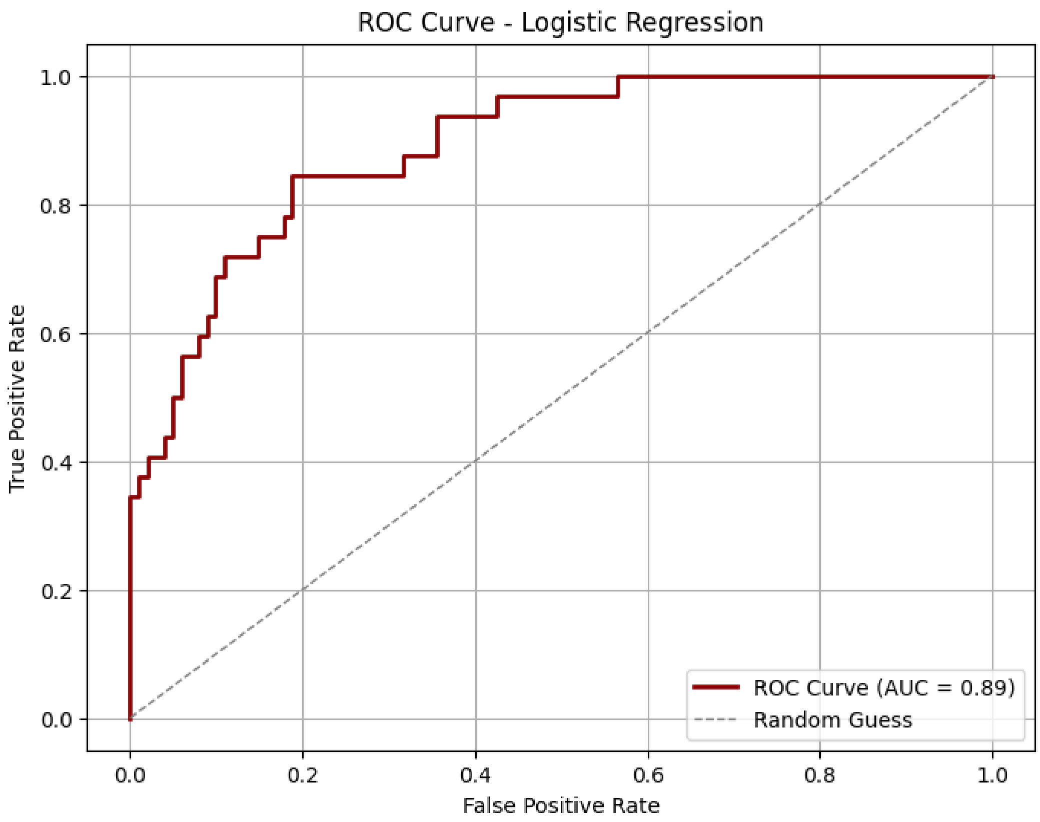

ROC Curve - Random Forest

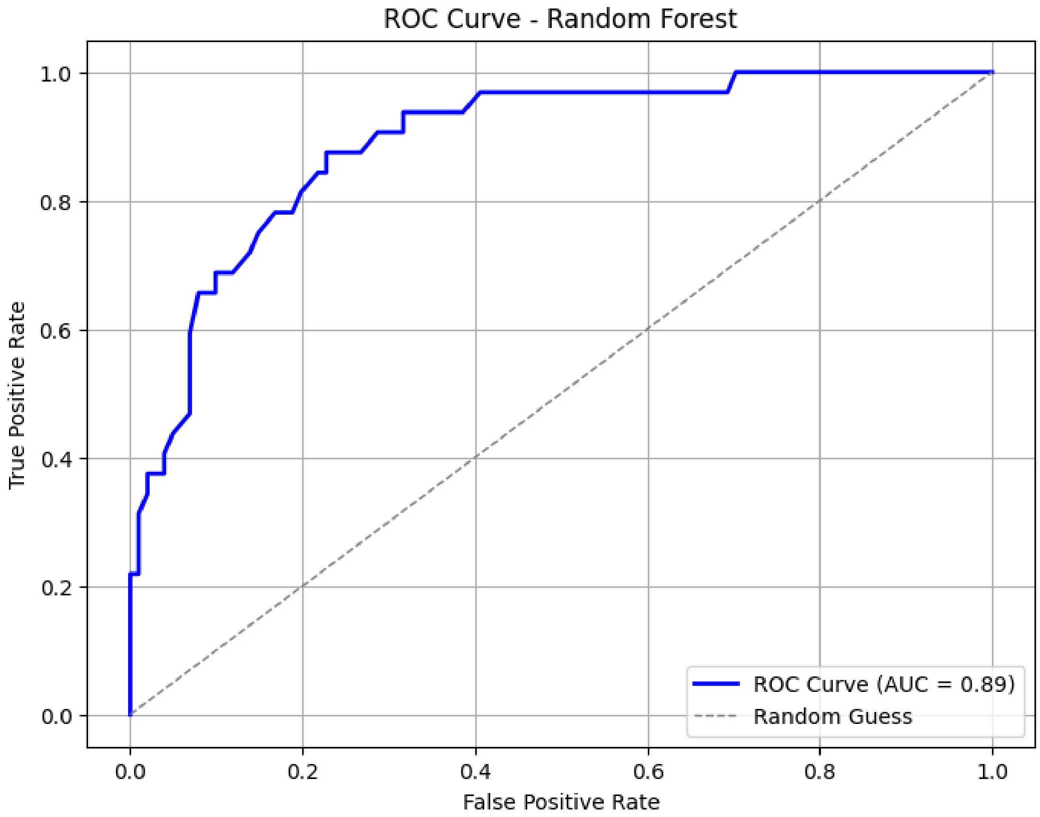

ROC Curve - Gradient Boosting

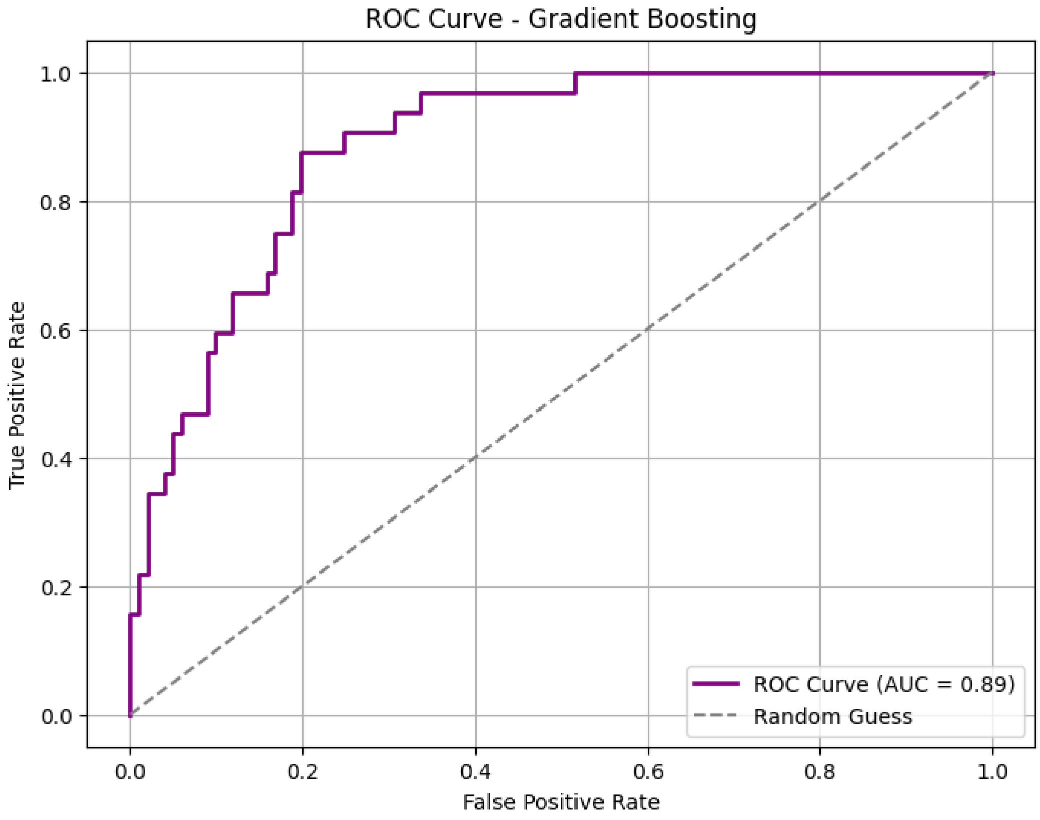

ROC Curve - Support Vector Machine

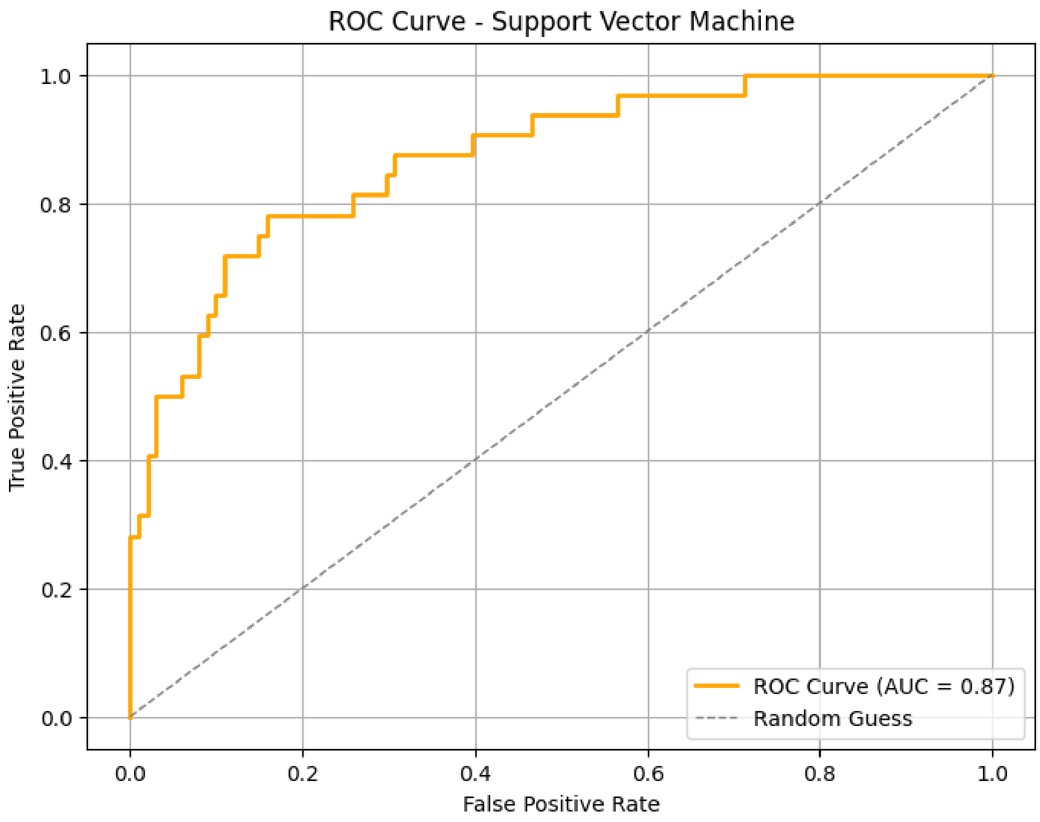

ROC Curve - K-Nearest Neighbors

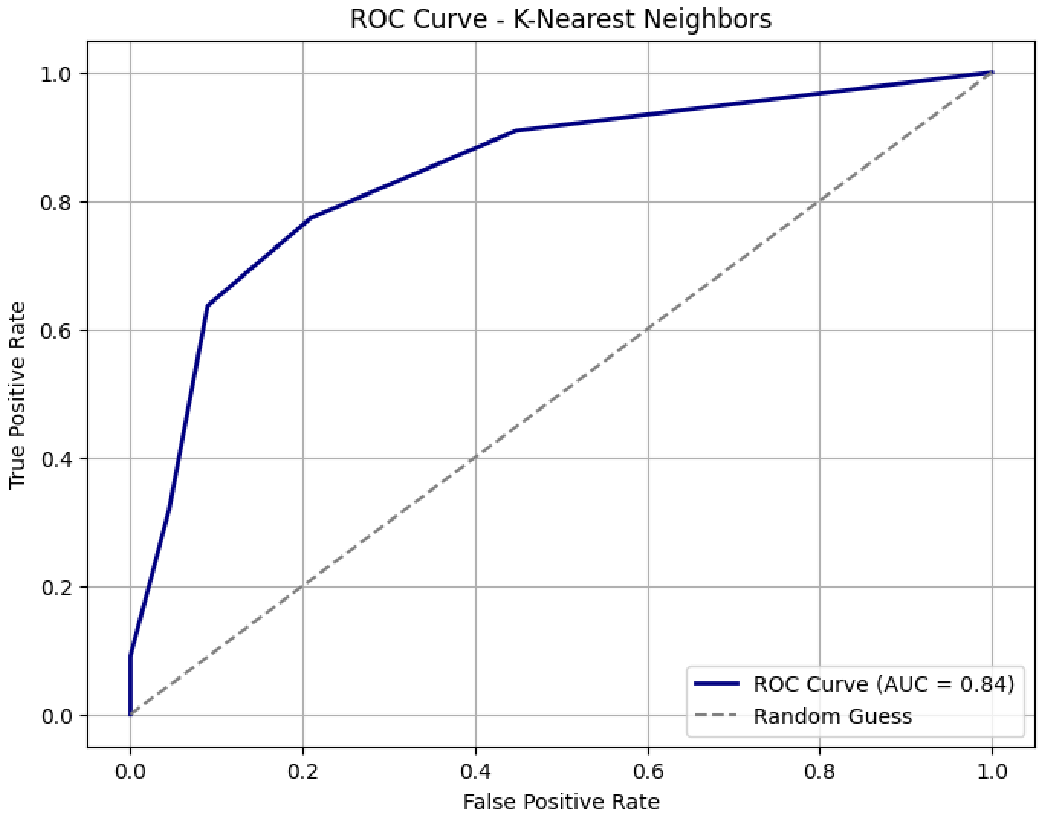

ROC Curve - FCNN Model

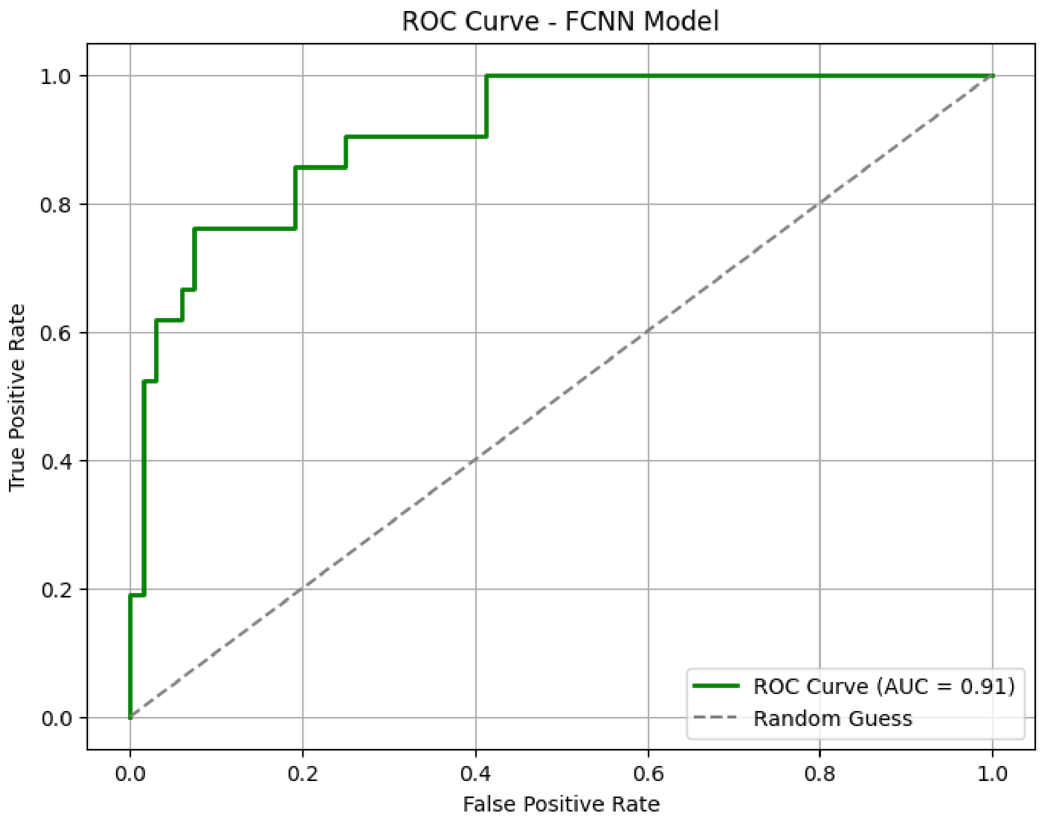

ROC Curve - Deep Neural Network

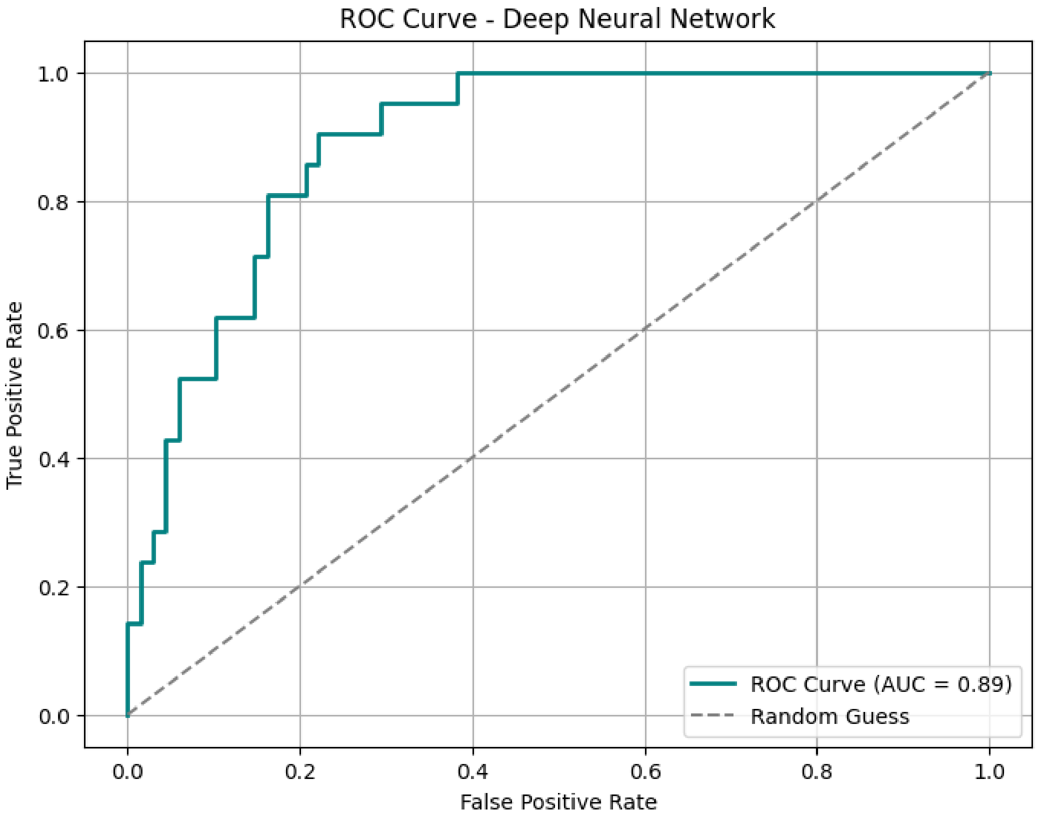

ROC Curve - RNN Model

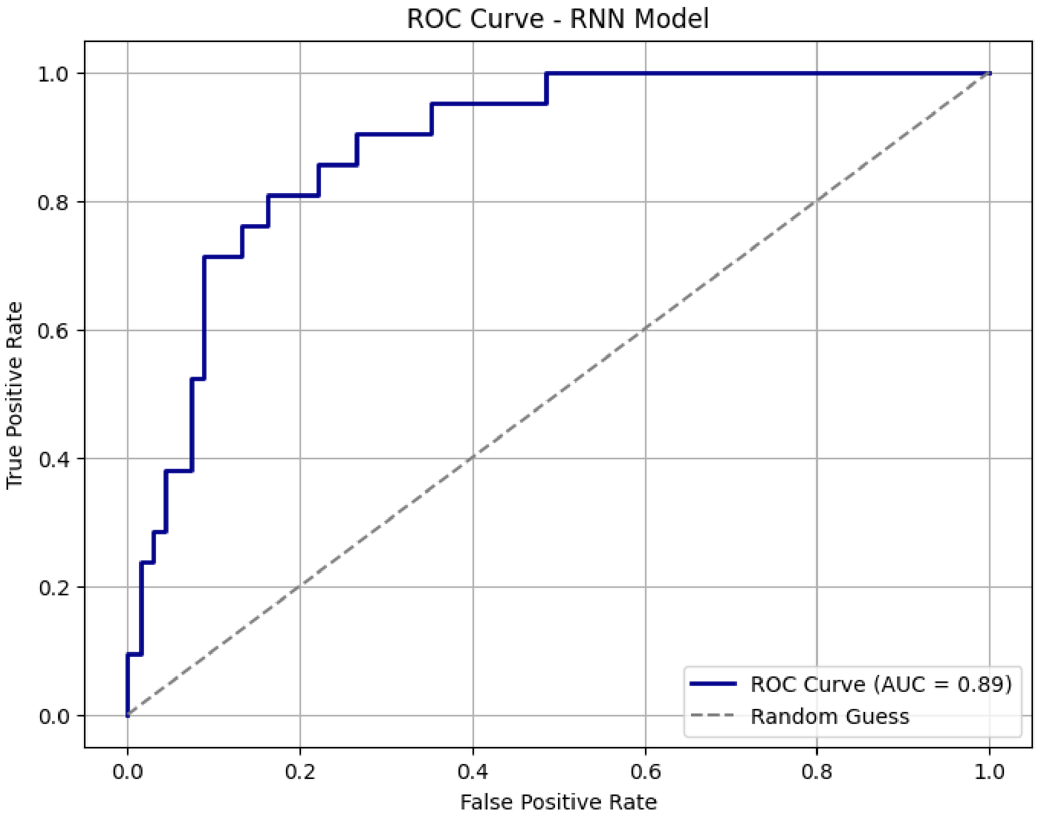

ROC Curve - LSTM Model

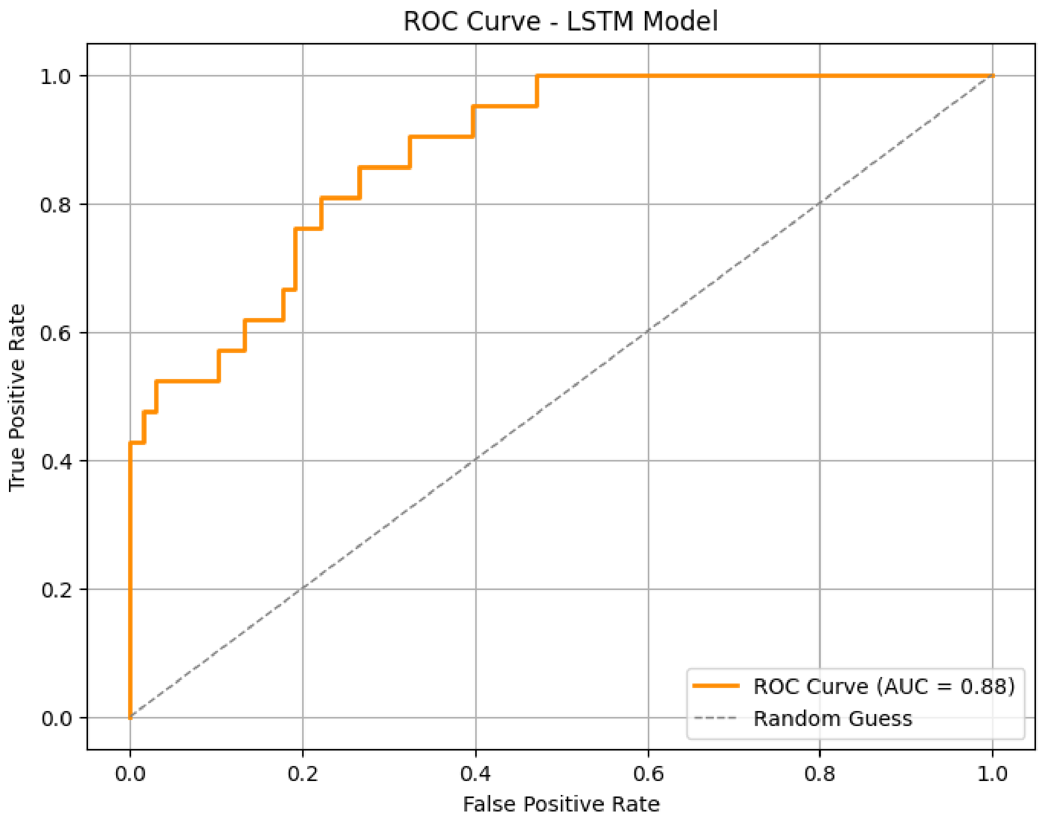

ROC Curve - Wide & Deep Model

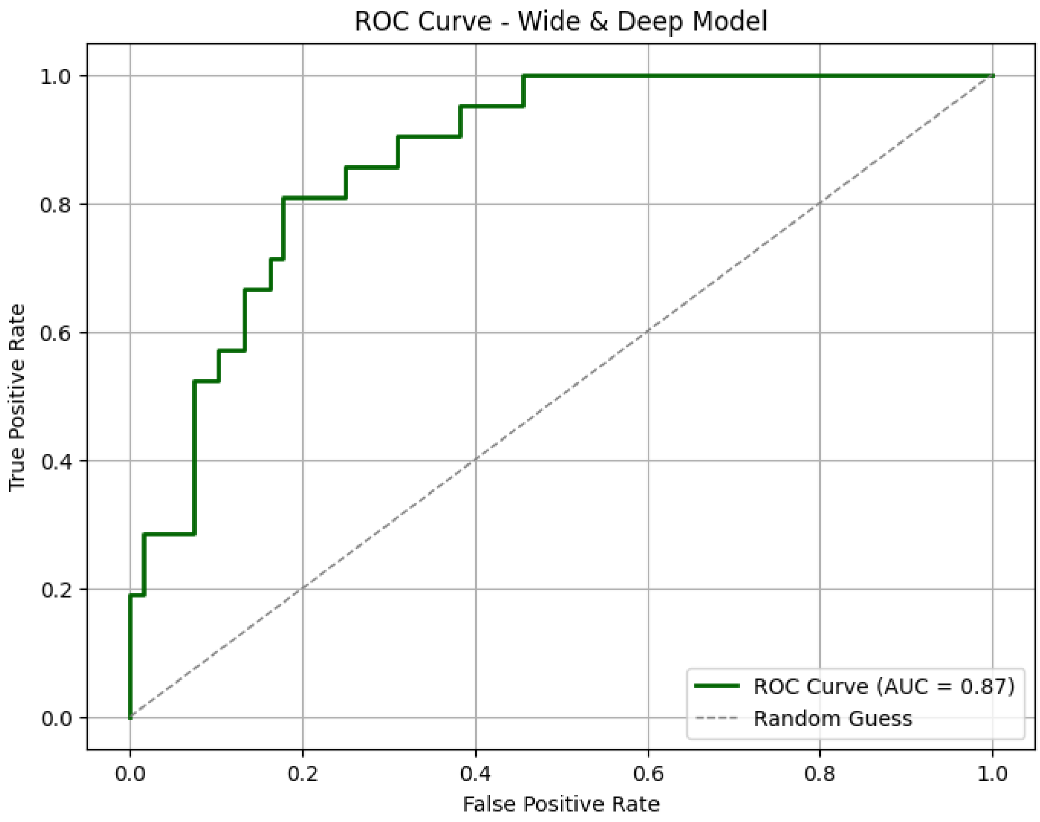

Supplement: Supplementary file 2 — Supplement 2. Receiver operating characteristic curves for the 10 traditional deep learning or machine learning models. [file emj-2025-00353-Supplement-2.pdf]
